# Supplementary material for: Identification of photoperiod-induced specific miRNAs in the adrenal glands of Sunite sheep (Ovis aries)
Source: Front Vet Sci. 2022 Jul 22;9:888207. doi: 10.3389/fvets.2022.888207 (PMC9354845; doi:10.3389/fvets.2022.888207)
Supplement: Supplementary file 1 [file Data_Sheet_1.ZIP › Supplementary file/miRDeep2 file.zip!/miRDeep2 file/Novel_Structure/LP42A2/Novel_197.pdf]

Provisional ID	: NC_019459.2_3852
Score total	: 2.6
Score for star read(s)	: -1.3
Score for read counts	: 0
Score for mfe	: 2.3
Score for randfold	: 1.6
Score for cons. seed	:
Total read count	: 244
Mature read count	: 244
Loop read count	: 0
Star read count	: 0

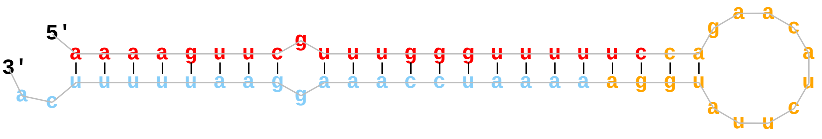

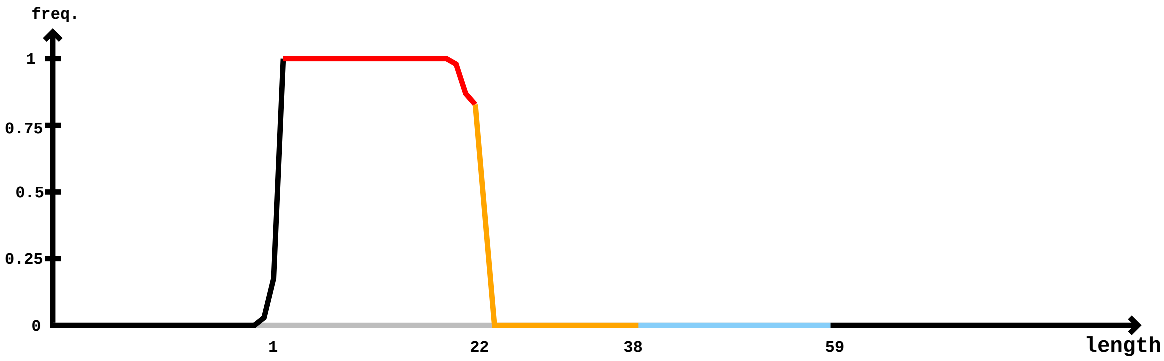

Mature **Star**

```
5'- ucaaauaauauuggguuggcugaaaaguucguuuggguuuuuccagaacaucuuauggaaaaauccaaaggaauuuuucagccaacacaauauuaacuuucacagcuuugu   -3'    exp
    .....(((((((.(((((((((((((((.(((((((((((((..........))))))))))))).))))))))))))))).))))))).......................   reads    mm    sample
    ....................ugaaaaguucguuuggguuuu......................................................................   2        0     seq
    ....................ugaaaaguucguuuggguuuuu.....................................................................   5        0     seq
    .....................Aaaaaguucguuuggguuu.......................................................................   5        1     seq
    .....................Aaaaaguucguuuggguuuu......................................................................   24       1     seq
    .....................gaaaaguucguuuggguuuu......................................................................   1        0     seq
    .....................gaaaaguucguuuggguuuuu.....................................................................   5        0     seq
    .....................gaaaaguucguuuggguuuuuc....................................................................   1        0     seq
    ......................aaaaguucguuuggguuuuuc....................................................................   127      0     seq
    ......................aaaaguucguuuggguuuuuA....................................................................   8        1     seq
    ......................aaaaguucguuuggguuuuucG...................................................................   2        1     seq
    ......................aaaaguucguuuggguuuuucA...................................................................   64       1     seq
```
